# Orthopaedic Consultation Versus No Consultation for Clavicle, Radial Head, and Proximal Humerus Fractures: Association with Post-Imaging Emergency Department Length of Stay

**DOI:** 10.3390/jcm15208071

**Published:** 2026-10-17

**Authors:** Lindsay V. Walsh, Jacob S. Borgida, Austin T. Gregg, Vinicius L. Craveiro, Lucy Zhao, Samantha Brown, Mark P. Cote, Robert K. Wagner, Ali Raja, Thuan V. Ly

**Affiliations:** 1 Department of Emergency Medicine, Mass General Brigham, Boston, MA 02114, USA; 2 Harvard Orthopaedic Trauma Initiative, Mass General Brigham, Boston, MA 02114, USA; 3 Department of Orthopaedic Surgery, Mass General Brigham, Boston, MA 02114, USA

**Keywords:** emergency department, length of stay, orthopaedics, trauma, clinical care protocol

## Abstract

**Background/Objectives**: To evaluate the association between orthopaedic consultation and Emergency Department (ED) length of stay among patients with clavicle, radial head, and proximal humerus fractures meeting criteria for nonacute management (discharge without orthopaedic consultation). **Methods**: All adult patients presenting to a single Level 1 trauma centre between 2019 and 2022 with a clavicle, radial head, or proximal humerus fracture that met criteria for nonacute management (discharge without orthopaedic consultation) were identified from an institutional database. Post-imaging ED length of stay, defined as time from radiograph completion to discharge or admission, was compared between patients discharged per protocol without orthopaedic consultation and those discharged after receiving orthopaedic consultation. A mixed-effects linear log regression model with orthopaedic surgery consult, age and sex as fixed effects and year of presentation as a random intercept was used to model the effect of not having an orthopaedic surgery consult on length of stay. A post hoc sensitivity analysis excluding patients who were admitted was performed. Results of the regression model are presented with corresponding 95% confidence intervals. **Results**: This study included 523 patients (average age 57 years, 58% female) with 115 clavicle, 269 proximal humerus, and 139 radial head fractures. Among patients meeting criteria for nonacute management, those who were discharged per protocol without orthopaedic consultation had a 59%, 56%, and 55% reduction in length of stay for clavicle, radial head, and proximal humerus fractures, respectively (all *p* < 0.001). Only 3/523 (0.6%) were admitted to orthopaedic surgery. Results were consistent in post hoc sensitivity analysis. **Conclusions**: Discharge without orthopaedic consultation among patients with clavicle, radial head, or proximal humerus fractures meeting criteria for nonacute management was associated with substantially shorter ED length of stay. Adherence to this protocol may aid in reducing overcrowding in the ED.

## 1. Introduction

Emergency Department (ED) overcrowding has remained a persistent barrier to timely and efficient patient care since the 1980s [1]. Despite the widespread recognition of this challenge, overcrowding continues to reach unprecedented levels nationwide, leading to delays in care, increased length of stay, and worse outcomes [2,3,4,5]. Patient volume and boarding, subspecialty consultations, and insufficient staffing are major contributors to overcrowding [6,7]. Due to its complexities, overcrowding requires practical clinical care protocols and the implementation of multidisciplinary changes.

Approximately one in five patients present to the ED with musculoskeletal complaints, many of which receive appropriate orthopaedic surgery consultation [8,9]. The proportion of appropriate orthopaedic consultations, and a smaller subset of unnecessary consults, varies based on injury severity, institutional protocols, and available resources [10]. A recent prospective study found that nearly 40% of orthopaedic consultations were inaccurate, leading to significant management changes in a majority of cases and an incomplete initial workup of patients [11]. These findings underscore the need for improved adherence to management protocols and structured consultation guidelines to optimise outcomes. Understanding the workflow of common orthopaedic injuries in the ED and the impact of orthopaedic consultation on length of stay and patient care can further elucidate potential solutions to ED overcrowding. Previous investigators have proposed structured treatment protocols for isolated displaced midshaft clavicle fractures to guide operative versus nonoperative management based on objective radiographic parameters [12]. However, these protocols focus on fracture-specific treatment decisions rather than ED consultation practices or operational outcomes. There remains limited evidence regarding standardised clinical care pathways for determining when orthopaedic consultation is necessary.

To reduce ED length of stay for clavicle, radial head, and proximal humerus fractures meeting criteria for nonacute management, the authors assessed a clinical care protocol that provides specific indications for orthopaedic consultation such as open fractures, skin tenting, fractures with dislocation, and neurovascular compromise. Fractures that did not meet criteria were not indicated for orthopaedic consultation (also termed “nonacute management”). The primary purpose of this study was to evaluate the association between orthoapedic consultation and ED length of stay among patients with clavicle, radial head, and proximal humerus fractures that met criteria for nonacute management by comparing patients discharged per protocol without orthopaedic consultation and those who were discharged after receiving orthopaedic consultation. We hypothesised that patients discharged without orthopaedic consultation would have shorter ED length of stay compared to those who received orthopaedic consultation.

## 2. Materials and Methods

After local IRB approval (Massachusetts General Hospital Protocol #2023P000179), all patients who presented to a single academic Level 1 trauma centre between 2019 and 2022 with a clavicle, radial head, or proximal humerus fracture were identified using International Classification of Diseases (ICD-10) codes S52.12, S42.0, and S42.2. Patients were excluded if evaluated at an outside institution for initial fracture evaluation, underwent polytraumatic injury or pathologic fracture, were under 18 years of age, or required evaluation by non-orthopaedic consultants during the indexed ED visit. Medical records and pertinent imaging were reviewed to determine (1) length of stay, defined as time elapsed from radiograph completion in the ED to time of discharge or admission as delineated in the medical record, and (2) whether the orthopaedic surgery service was consulted during each patient encounter. Data collection and medical record review were performed by two research assistants under the supervision of fellowship-trained orthopaedic trauma surgeons. Reviewers were not blinded to length of stay, and inter-rater agreement was not formally assessed. Medical records and radiographic images were retrospectively reviewed to determine whether each patient met criteria for nonacute management according to the ED orthopaedic consult protocol (Appendix A). For radial head fractures, consultation was indicated for open fracture, skin tenting, neurovascular compromise, comminution, or dislocation. For clavicle fractures, consultation was indicated for open fracture, skin tenting, neurovascular compromise, or ≥2 cm displacement and shortening. For proximal humerus fractures, consultation was indicated for open fracture, skin tenting, neurovascular compromise, or humeral head dislocation. Patients without these fracture-specific indications were considered eligible for nonacute management. Eligibility for nonacute management was based on the absence of indications for urgent orthopaedic consultation rather than fracture complexity or the potential need for subsequent operative treatment. During the study period, the decision to obtain an immediate orthopaedic consultation was made at the discretion of the treating ED provider and was not prospectively standardised by the study team. For purposes of this retrospective analysis, the predefined institutional protocol criteria described above were confirmed during medical record and radiographic review to determine whether each patient was eligible for nonacute management. If nonacute criteria were met, an orthopaedic surgery consult in the ED was deemed clinically unnecessary. The clinical care protocol is standard-of-care for the study institution and used as instructional material. Patients with these injuries who met criteria for nonacute management were instructed to follow up in the orthopaedic clinic within one week, regardless of whether orthopaedic surgery was consulted during the initial ED encounter.

Patients were grouped according to fracture type, and analyses were performed separately for each group. Data abstracted from the medical record included an orthopaedic consultation, admission to the orthopaedic surgery service, and whether subsequent surgery (inpatient or outpatient) was required. The primary outcome for patients included in this study was length of stay. Secondary outcomes were the percentage of patients requiring admission and the percentage requiring inpatient or outpatient surgery.

### Statistical Analysis

Descriptive statistics were calculated to characterise the study cohort, including mean and standard deviation for continuous variables, and frequency and proportion for categorical variables. The distribution of time spent in the ED was graphically examined and found to be right-skewed. However, the log of time spent in the ED produced a normal distribution. We analysed length of stay time in its log form as the results provide the percent increase in time associated with an orthopaedic consult. The difference in length of stay across days of the week and year of presentation was assessed using one-way ANOVA and boxplots. There was a significant difference in length of stay across years (*p* = 0.022) with an increasing trend on inspection of boxplots. We used a mixed-effects linear log regression model with orthopaedic surgery consult, age and sex as fixed effects and year of presentation as a random intercept to model the effect of not having an orthopaedic surgery consult on length of stay. A post hoc sensitivity analysis excluding patients who were admitted was performed. Results of the regression model are presented with corresponding 95% confidence intervals. No a priori sample size calculation was performed, as all patients meeting the eligibility criteria within the study timeframe were included. Statistical significance was established at *p* < 0.05. All statistical analyses were performed using R version 4.0 (R Foundation for Statistical Computing, Vienna, Austria) with the tidyverse, gtsummary, lme4, lmerTest, and broom.mixed packages.

## 3. Results

**Figure 1:**
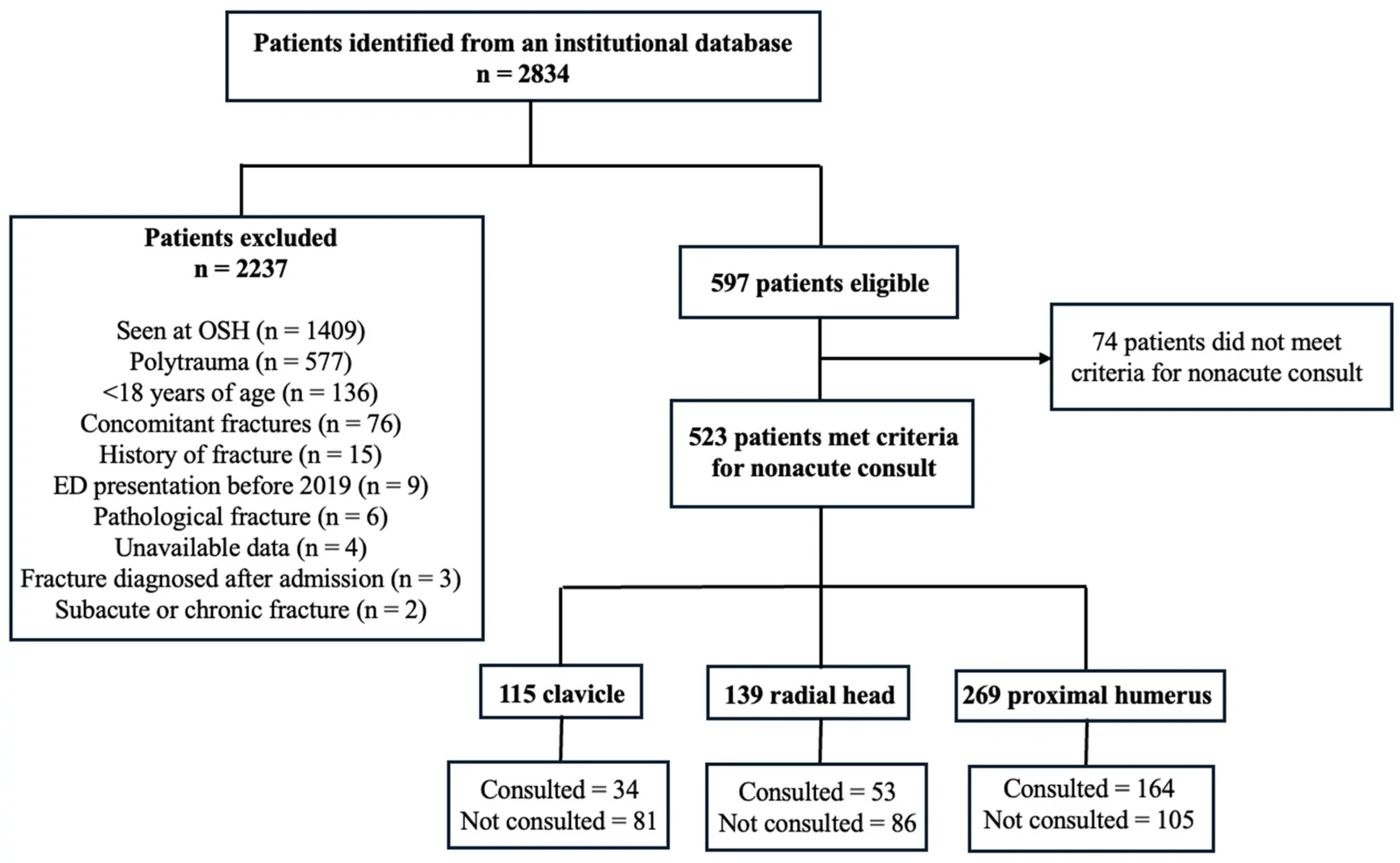
Flow diagram for patients identified, screened, and included.

The institutional database search returned 2834 patients, of whom 523 met the nonacute consultation criteria (Figure 1). The mean age of patients was 57 ± 20 years, and 300 patients (58%) identified as female. The overall ED average length of stay was 5.0 ± 8.3 h (Table 1).

### 3.1. Clavicle Fractures

**Figure 2:**
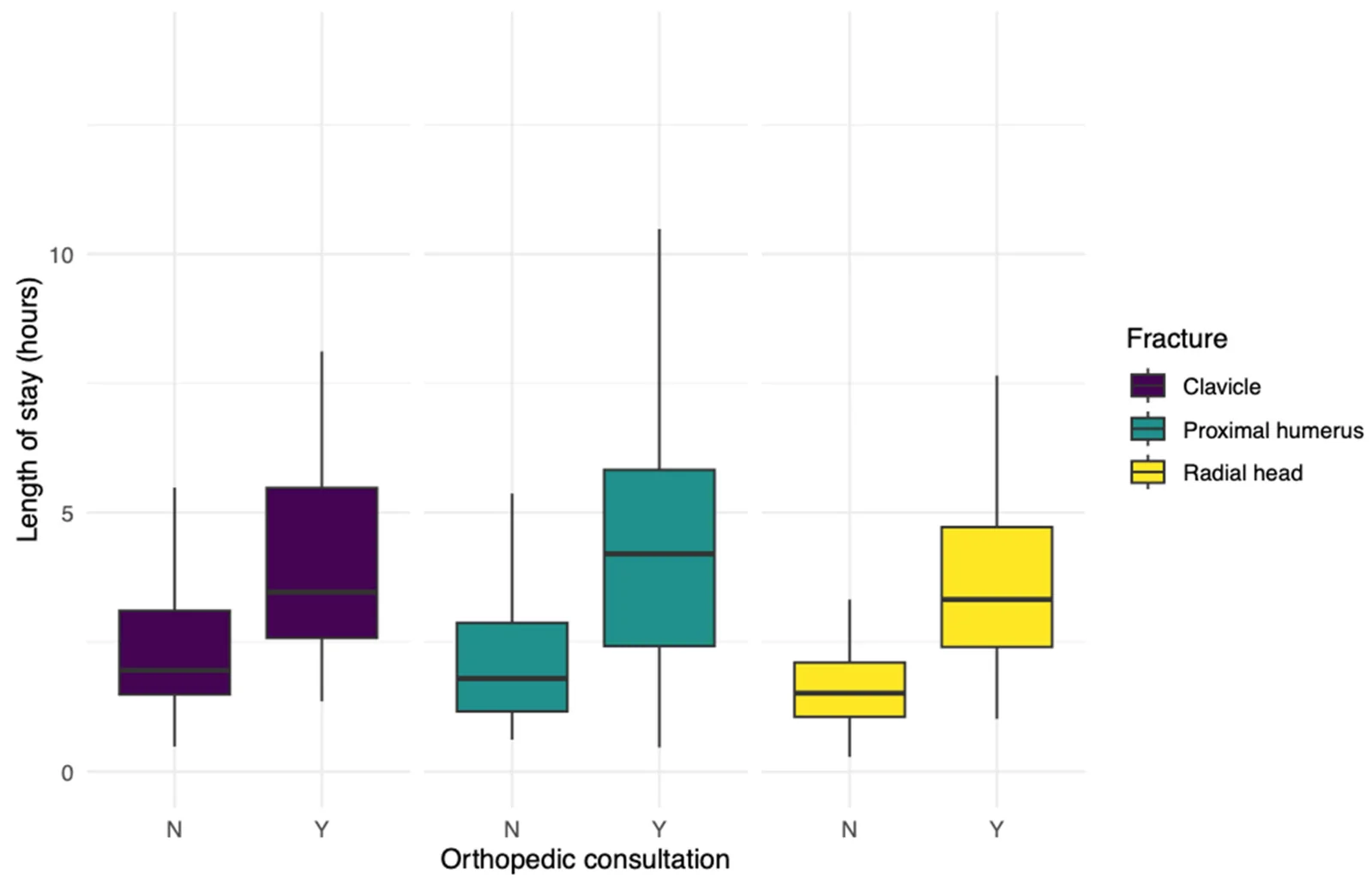
Boxplot displaying length of stay in the Emergency Department by orthopaedic surgery consultation.

In total, 115 patients with a clavicle fracture met the criteria for nonacute management. The mean age was 42 ± 28 years, and 55 patients (48%) identified as female. Orthopaedic surgery was consulted in 34 cases (30%), and length of stay was on average 6.4 h less when patients were discharged per protocol without orthopaedic consultation (Table 2 and Figure 2). Specifically, patients who did not receive an orthopaedic consult had a 59% reduction in length of stay (Table 3). Thirty patients (26%) were admitted, of which 16 (53%) were to non-orthopaedic inpatient units for further evaluation or management of medical needs and 14 (47%) to ED observation. Five patients (4.3%) underwent surgery in outpatient settings.

### 3.2. Radial Head Fractures

In total, 139 patients with a radial head fracture met the criteria for nonacute management. The mean age was 47 ± 25 years, and 99 patients (71%) identified as female. Orthopaedic surgery was consulted in 53 cases (38%), and length of stay was on average 2.1 h less when patients were discharged per protocol without orthopaedic consultation (Table 2 and Figure 2). Specifically, patients who were not consulted by orthopaedic surgery had a 56% reduction in length of stay (Table 3). Five patients (3.6%) were admitted, of which three (60%) were to non-orthopaedic inpatient units and two (40%) to the ED observation unit. Three patients (2.2%) underwent nonacute surgery in an outpatient setting.

### 3.3. Proximal Humerus Fractures

In total, 269 patients with a proximal humerus fracture met the criteria for nonacute management. The mean age was 65 ± 16 years, and 202 patients (75%) identified as female. Orthopaedic surgery was consulted in 164 cases (61%), and length of stay was on average 4.3 h less when patients were discharged per protocol without orthopaedic consultation (Table 2 and Figure 2). Patients who were not consulted by orthopaedic surgery had a 55% reduction in length of stay (Table 3). Eighty-two patients (30%) were admitted, of which 3 (3.7%) were to orthopaedic surgery, 24 (29%) to non-orthopaedic inpatient units, and 55 (67%) to ED observation. Forty-two patients (16%) underwent nonacute surgery in the outpatient setting. Of the patients admitted to orthopaedic surgery, two (0.7%) had inpatient surgery: one patient had a displaced comminuted fracture and the other had a >100% displaced fracture with shortening and mild angulation. The one patient admitted who did not undergo operative fixation was due to a delay in discharge approval for return to a skilled nursing facility.

### 3.4. Post Hoc Sensitivity Analysis

In the post hoc sensitivity analysis restricted to patients who were not admitted, the association between absence of orthopaedic consultation and shorter ED length of stay remained significant across all three fracture groups, consistent with the primary analysis (Appendix B).

## 4. Discussion

At our institution, a formal clinical care protocol for orthopaedic consultation was implemented into the ED care pathway for clavicle, radial head, and proximal humerus fractures after an excess of non-emergent orthopaedic consultations was observed. The present study found that adherence to this clinical care protocol was associated with significantly shorter ED length of stay. Across all three fracture groups, patients with care plans that followed the formal consultation protocol had shorter ED length of stay by 59%, 56%, and 55% for clavicle, radial head, and proximal humerus fractures, respectively. These findings demonstrate an association between adherence to formal clinical care protocols for orthopaedic injuries and shorter ED length of stay.

### 4.1. Length of Stay

Extended length of stay in the ED has been shown to reduce patient satisfaction, delay care, and increase healthcare expenditures [13,14,15]. It also disrupts patient flow within the ED, leading to downstream effects of higher morbidity rates, increased adverse events, unnecessary follow-up appointments, and undue financial burdens [16,17,18]. Moreover, prolonged length of stay contributes to ED overcrowding, which negatively affects the performance and wellbeing of healthcare workers and associated staff [19]. These challenges highlight the critical need for the appropriate utilisation of subspecialty consultation. Although few studies have specifically examined the impact of orthopaedic consultation on ED length of stay, Bashkin et al. reported that patients admitted to the orthopaedic department had the longest mean ED length of stay among six admitting departments (382 min) [20]. Our study found that across all three fracture groups, patients with care plans that followed the formal clinical care protocols had significantly reduced ED length of stay.

### 4.2. Appropriate Consultations

The results of this study highlight the necessity of appropriate and accurate orthopaedic consultations in the ED. Notably, orthopaedic surgery was consulted nearly half (48.1%) of the time for fractures that did not require orthopaedic intervention by institutional clinical care protocols. This is consistent with other studies that have found similarly high rates of unnecessary orthopaedic consultations [11]. These findings emphasise the importance of implementing and adhering to formal consultation clinical care protocols. Mears et al. previously demonstrated that a system for musculoskeletal care of select injuries significantly decreased the rate of orthopaedic consultation without adverse effects on patient care or safety [21]. Biz et al. also described an institutional protocol for isolated displaced midshaft clavicle fractures that used objective radiographic criteria to guide operative versus nonoperative treatment [12]. Although both studies support standardised approaches to fracture management, the present protocol differs in both scope and purpose. Rather than directing definitive treatment for a single fracture type, our protocol focuses on identifying clavicle, radial head, and proximal humerus fractures that do not require urgent orthopaedic consultation. By evaluating the impact of protocol adherence on ED length of stay, the present study extends the application of standardised clinical pathways from fracture-specific treatment algorithms to ED consultation practices and resource utilisation. Emergent orthopaedic surgery consultation was indicated for fractures that are open, dislocated, and neurovascularly compromised. Of the eligible patients, 524/598 (88%) met criteria for nonacute management, showing that the proposed clinical care protocol applies to the majority of clavicle, radial head, and proximal humerus fractures. However, over half of the patients who met the requirements for nonacute management in the present study still received orthopaedic consultation, leading to a significant increase in length of stay.

The present protocol is also consistent with broader trends toward standardised musculoskeletal care pathways that aim to improve efficiency while maintaining high-quality patient care. Virtual fracture clinic models have demonstrated that protocol-driven management of appropriately selected, stable fractures can safely reduce in-person fracture clinic visits, lower healthcare costs, and maintain high levels of patient satisfaction, while fracture liaison services have shown the value of standardised care pathways in improving care coordination and reducing avoidable complications following fragility fractures [22,23,24,25]. More recently, standardised orthopaedic triage systems have further demonstrated the potential to reduce unnecessary consultations while preserving appropriate specialist involvement in proximal humerus fractures [26]. Although these initiatives target different patient populations and stages of care, they share the common objective of delivering high-quality, protocol-driven musculoskeletal care while improving healthcare efficiency and resource utilisation. The present protocol builds on these broader care models by applying standardised triage principles to ED orthopaedic consultation for common upper extremity fractures, demonstrating that appropriate consultation practices can substantially reduce ED length of stay.

### 4.3. Percent of Patients Requiring Admission or Inpatient Surgery

Formal clinical care protocols are critical for optimising patient care decisions for fractures that meet the criteria for nonacute management. Among all three fracture groups, 22% of patients were admitted to the hospital, yet only one patient (0.2%) was admitted to the orthopaedic surgery service. Importantly, classification as appropriate for nonacute management did not preclude subsequent operative treatment, but rather indicated that acute orthopaedic consultation in the ED was not required under the institutional protocol. These findings suggest that opportunities may exist to optimise resource utilisation through greater adherence to established orthopaedic consultation criteria [10]. Silverman et al. analysed 13,777 orthopaedic surgery consults in the ED and found that 60% were for fractures; however, they did not report on the percentage of patients subsequently undergoing inpatient surgical intervention [10]. Further research is needed to clarify which orthopaedic consults may require inpatient surgical intervention, in order to better inform triage strategies. The results of this study can serve as a foundation for provider education, focusing on recognising fractures appropriate for nonacute management and applying the clinical care protocols effectively. Moving forward, the clinical care protocol for clavicle, radial head, and proximal humerus fractures meeting criteria for nonacute management will be integrated into the institution’s electronic health record (EHR) system. This integration will include decision-support tools, such as automated prompts and management recommendations, to assist ED providers. This initiative may help standardise consultation practices and improve ED efficiency, although further prospective evaluation is needed to determine its impact on ED length of stay and patient outcomes.

### 4.4. Limitations

There are limitations to this study. First, patients presenting at outside hospitals, with polytraumas, or pathologic fractures were excluded. Strict exclusion criteria were necessary to isolate the impact of orthopaedic consultation on length of stay. Additionally, length of stay was measured from radiograph completion to discharge or admission rather than from ED arrival; therefore, the reported values do not capture time from ED arrival to completion of imaging. Second, patients were identified using ICD-10 codes which can be subject to miscoding. To minimise this impact, EHRs of all consecutive patients were reviewed. Third, pathological fractures were not included as an indication for orthopaedic consultation in the ED. Patients who have sustained pathologic fractures or those admitted to non-orthopaedic surgery services often have more complex comorbidity profiles requiring additional workup and care coordination, which may over-report the length of stay findings in this study. To address these concerns, patients with multiple consulting services were excluded. Future clinical care protocols should evaluate the relative merit of including pathological fractures as an indication for orthopaedic consultation in the ED. Additionally, this was a retrospective, single-centre observational study, and causality cannot be established. Additionally, confounding by indication may have influenced the observed association, as patients for whom treating clinicians had greater diagnostic or management uncertainty may have been more likely to receive orthopaedic consultation and may also have had longer ED stays independent of the consultation itself. Although the statistical model was adjusted for year of presentation, residual confounding from unmeasured temporal changes in ED workflow, staffing, institutional organisation, patient volume, or other factors such as degree of fracture displacement, comorbidity burden, arrival time, and ED census may have contributed to the observed differences in length of stay independent of the clinical care protocol. Further, this study focused primarily on ED length of stay and was not designed to evaluate other measures of care quality, including delayed diagnoses, unplanned ED revisits, patient satisfaction, adherence to outpatient follow-up, complications, or functional outcomes. Additionally, the study did not assess total healthcare utilisation or cumulative patient time associated with the initial ED encounter and subsequent outpatient orthopaedic follow-up, and therefore cannot determine whether the observed reduction in ED length of stay translates to reduced overall healthcare utilisation or patient time. Because this study was conducted at a single academic Level I trauma centre with immediate access to orthopaedic specialists, these findings may not be generalizable to smaller hospitals, community EDs, or healthcare systems with different resources, consultation practices, or referral pathways. Future studies should evaluate these outcomes to further characterise the safety and effectiveness of nonacute management protocols. Lastly, our results may underestimate the proportion of patients who received surgical fixation for fracture if treated at an outside hospital after presenting to the ED.

## 5. Conclusions

Discharge without orthopaedic consultation among patients with clavicle, radial head, or proximal humerus fractures meeting criteria for nonacute management was associated with substantially shorter ED length of stay. Implementation and adherence to an ED clinical care protocol may aid in reducing overcrowding.

## Figures and Tables

**Table 1:** Descriptive statistics for patients presenting to the Emergency Department with clavicle, radial head, and proximal humerus fractures.

Variable	
Female	301 (58%)
Age, years	57 (±20)
Orthopaedic consultation	
Yes	251 (48%)
No	272 (52%)
Length of stay, hours	5.0 (±8.3)
Fracture	
Clavicle	115 (22%)
Proximal humerus	269 (51%)
Radial head	139 (27%)

Values given as mean (standard deviation) or frequency (proportion).

**Table 2:** Length of stay in the Emergency Department by orthopaedic surgery consultation.

Fracture	Orthopaedic Surgery Consultation, Length of Stay	LOS, Hours	No Orthopaedic Surgery Consultation, Length of Stay	LOS, Hours	Mean Difference (95% CI)
Clavicle	34 (30%)	10.1 (12.4)	81 (70%)	3.7 (5.1)	6.4 (3.2–9.6)
Radial head	53 (38%)	3.8 (2.0)	86 (62%)	1.7 (1.1)	2.1 (1.5–2.6)
Proximal humerus	164 (61%)	7.7 (10.9)	105 (39%)	3.4 (7.6)	4.3 (1.9–6.7)

Length of stay values are means with standard deviation and differences are mean differences with 95% confidence intervals.

**Table 3:** Mixed-effects linear log regression analysis of adequately using the nonacute management protocol for clavicle, radial head, and proximal humerus fractures adjusting for age and sex as fixed effects and including year of presentation as the random intercept; 95% CI: 95% confidence interval; Exp (Coefficient): exponentiated regression coefficient, which provides the relative model-based change in length of stay without an orthopaedic surgery consultation.

Fracture	Coefficient	95% CI	*p*-Value	Exp (Coefficient)	Relative Change in Length of Stay
Clavicle	−0.88	−1.23 to −0.53	<0.001	0.41	59% reduction
Radial head	−0.82	−0.99 to −0.63	<0.001	0.44	56% reduction
Proximal humerus	−0.79	−1.01 to −0.57	<0.001	0.45	55% reduction

## Data Availability

Data is available upon reasonable request.

## Abbreviations

The following abbreviations are used in this manuscript:

ED	Emergency Department

## Appendix A

**Table A1:** Orthopaedic surgery consult criteria (July 2022).

Fracture/Injury	Radiographic Notes	Call Consulting Service If	Radiographic Studies	Treatment	Follow-Up
Clavicle	Medial/middle/distal third Z-deformity?	Open Fracture Tenting Skin Neurovascular Compromise 2cm displacement and shortened	3 View Clavicle - Upright AP View - Upright Cephalad View - Panoramic	Sling NWB Reclining position to decrease pain	1 Week
Proximal Humerus	NEER Criteria - Location: surgical neck, anatomic neck, greater tuberosity, lesser tuberosity - Dislocation: any part of head in joint? - # of parts? (2, 3, >3)	Open Fracture Tenting Skin Neurovascular Compromise Humeral head dislocation	3 View Shoulder Series - AP - Axillary - Scapular Y	Sling or Shoulder Immobiliser NWB	1 Week
Radial Head and Neck Note: Consider compartment syndrome with all forearm fxs	Displaced? Articular involvement? Comminution? Dislocated? Galeazzi: Mid shaft radial fracture w/ulnar head dislocation Terrible triad: Elbow dislocation Coronoid fracture Radial head fracture	Open Fracture Tenting Skin Neurovascular Compromise Comminuted Dislocated	3 View Elbow AP/Lateral/Radiocapitellar View	Sling NWB	1 Week

## Appendix B

**Table A2:** Post hoc sensitivity analysis showing relative length in stay for non-admitted patients. 95% CI: 95% Confidence Interval, Exp (Coefficient): Exponentiated regression coefficient, which provides the relative model-based change in length of stay without an orthopaedic surgery consultation.

Fracture	Coefficient	95% CI	*p*-Value	Exp (Coefficient)	Relative Change in Length of Stay
Clavicle (*n* = 85)	−0.52	−0.88 to −0.17	0.005	0.59	41% reduction
Radial head (*n* = 134)	−0.81	−0.99 to −0.62	<0.001	0.45	55% reduction
Proximal humerus (*n* = 187)	−0.80	−1.00 to −0.61	<0.001	0.45	55% reduction
